# The ascorbate transporter SVCT2 to target microglia-dependent inflammation

**DOI:** 10.18632/oncotarget.22306

**Published:** 2017-11-07

**Authors:** Camila C. Portugal, Renato Socodato, João B. Relvas

**Affiliations:** Camila C. Portugal: Instituto de Investigação e Inovação em Saúde and Instituto de Biologia Molecular e Celular (IBMC), Universidade do Porto, Porto, Portugal

**Keywords:** caveolin-1, NF-kB, Src kinase, ROS, neuroinflammation

Inflammation is an immune response triggered by several factors, including infectious pathogens, tissue injury and oxidative stress, with the purpose of restoring tissue homeostasis. In the central nervous system (CNS), inflammatory reactions differ from those of other organs because the reduced permeability of both blood brain barrier and cerebrospinal fluid barrier microvessels limit the recruitment of peripheral cells involved in innate and adaptive immune responses [1], positioning microglia as the major immune effector cells regulating brain inflammation.

Microglia are a small and distinct population of cells of mesenchymal origin, which vary from macroglia in function, morphology and gene expression profile. In a healthy brain, microglia actively patrol the parenchyma, monitor the functioning of synapses and control neuronal connectivity. Upon trauma, ischemia or infection, microglia mediate immune responses by regulating several branches of the inflammatory processes [2]. During this process, they assume an “amoeboid” cell morphology due to process retraction, become motile, migrate to misbalanced areas within the neuronal parenchyma, proliferate and phagocyte cell debris and/or damaged neurons [2]. They also enhance the expression of class II MHC and secrete a plethora of factors such as inflammatory cytokines, chemokine, glutamate, RNS and ROS [2].

Vitamin C is one of the most important low molecular weight antioxidants in the body. It is present in high concentrations in the CNS and is required for normal brain functioning [3]. Vitamin C is found in two forms, the oxidized form, dehydroascorbate (DHA), and the reduced form, ascorbate. DHA is taken up by glucose transporters (GLUT) [3] while ascorbate is taken up by the Sodium Vitamin C co-Transporter - SVCT (Slc23) [3]. SVCT2 is a glycoprotein with 12 transmembrane domains that transports ascorbate in a sodium-dependent manner [3], with potential N-glycosylation sites in the extracellular loop between transmembrane segments three and four [3]. This transporter can be regulated by multiple signaling pathways including PKA [3], and PKC [3].

SVCT2 transports ascorbate into CNS cells [3], including microglia [4]. Although cells use distinct transport mechanisms to take up ascorbate and DHA, only ascorbate has antioxidant properties, which is critical for the maintenance of the redox balance in the healthy and diseased CNS [5]. Because ascorbate directly shapes the redox balance, modulation of ascorbate bioavailability, by regulating SVCT2 expression, could potentially regulate microglia-associated inflammation.

**Figure 1:**
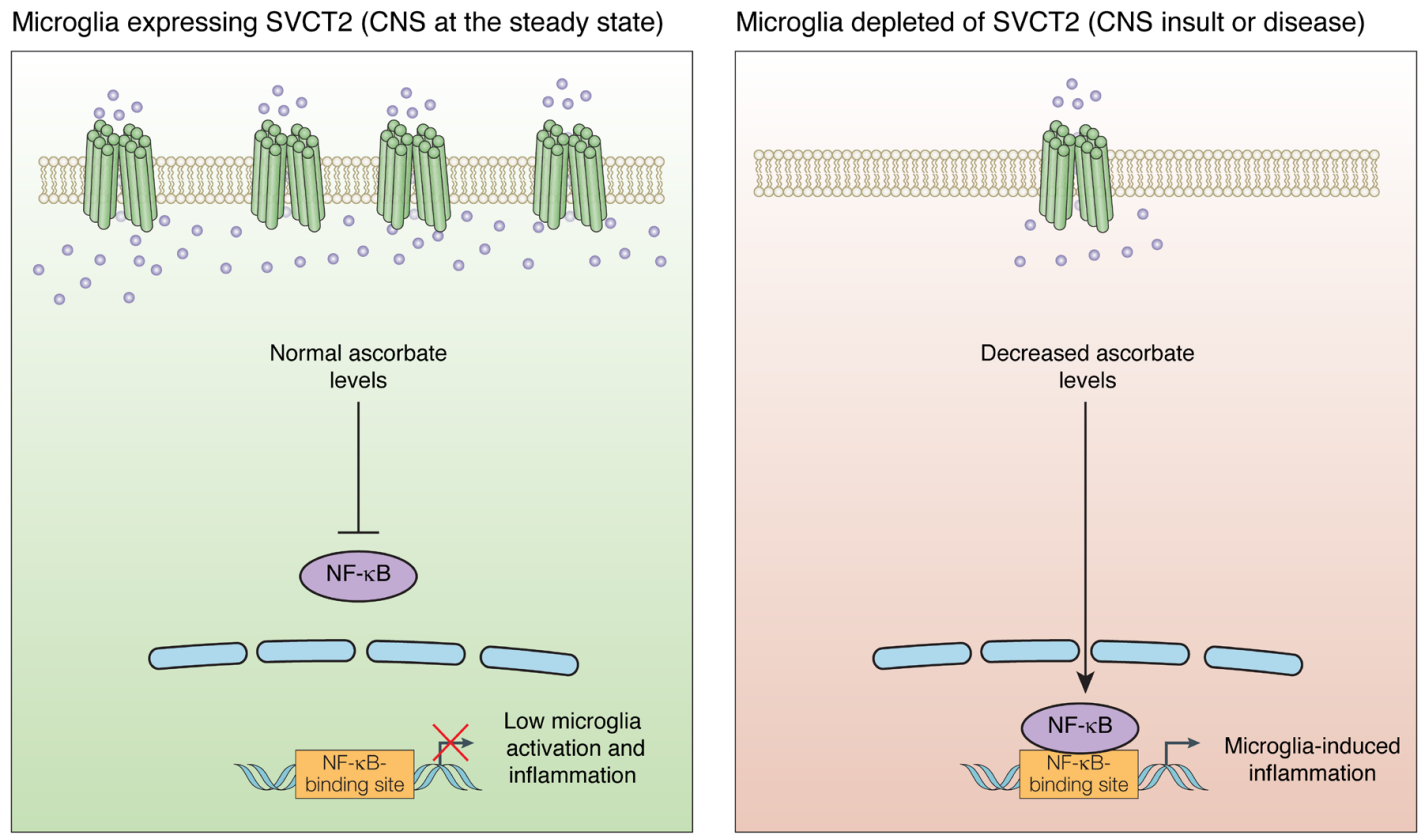
SVCT2 regulates microglial pro-inflammatory activation Left panel: Normal functioning of SVCT2 in non-stimulated microglia. SVCT2 is expressed in the plasma membrane and concentrates ascorbate in the intracellular space. Right panel: After a pro-inflammatory challenge, microglia reduces the expression of the SVCT2 in the plasma membrane (for the detailed signaling pathway involved in this process, please see [4]), resulting in decreased ascorbate uptake, which disrupts ascorbate homeostasis and activates microglia in a NF-κB-dependent manner. Therefore, decreasing ascorbate uptake in microglia, leads to ineffective inhibition of NF-κB, which triggers the production of pro-inflammatory mediators, such as TNF, IL-1β, IL-6 and NO.

In our recent work [4], we demonstrated that SVCT2 was responsible for ascorbate homeostasis in microglia and also that inducing CNS inflammation led to internalization and degradation of this transporter in microglia *in vivo* [4]. We also characterized a specific signaling pathway involved in SVCT2 internalization/degradation in microglia and showed that it involves inflammation-mediated Src activation, Src-dependent caveolin-1 phosphorylation and consequent SVCT2 degradation in the lysosome [4]. Bypassing the pro-inflammatory stimulation and decreasing SVCT2 expression, was sufficient to trigger pro-inflammatory activation of microglia, a phenotype corroborated *in vivo* using SVCT2-deficient mice and in human microglia depleted of SVCT2 [4]. Overexpressing SVCT2, preventing SVCT2 internalization or ascorbate treatment abrogated microglia pro-inflammatory polarization [4], suggesting that SVCT2 downregulation and consequent decrease of ascorbate uptake is necessary and sufficient for classical microglia activation (Figure 1).

We showed that depleting SVCT2 in microglia activates NF-κB [4] (Figure 1), a key step in the induction of pro-inflammatory activation in microglia [6]. NF-κB can be activated by direct application of oxidizing agents, like H_2_O_2_ [6] and be inhibited by ascorbate [7]. Accordingly, we showed that the reduction in the ascorbate uptake by decreasing SVCT2 expression activates NF-κB (Figure 1), inducing the production of pro-inflammatory mediators such as TNF, IL-1β and IL-6 and iNOS [4].

In line with this, deficits of vitamin C in the brain have been observed in different neurological conditions and disorders, including Parkinson´s and Alzheimer´s disease, in which microglia pro-inflammatory activation influences their onset and/or progression. Furthermore, when the APP/PSEN1^+^ mouse model for Alzheimer`s disease is crossed with SVCT2-deficient mice, thereby decreasing the ascorbate content in the brain, there was a marked increase in their cognition deficits, amyloid accumulation and oxidative stress [8].

Having demonstrated a critical role for SVCT2 and ascorbate uptake in regulating microglia homeostasis, it is tempting to speculate that in neurological conditions with a strong neuroinflammatory component, modulation of SVCT2 might constitute an attractive strategy for restoring microglia homeostasis and promoting neuronal viability.
